# Analysis of Axin2 expression and function in murine models for pancreatic cancer

**DOI:** 10.1186/s13578-016-0116-4

**Published:** 2016-08-22

**Authors:** Dietmar Zechner, Tim Kroemer, Ann-Christin Albert, Maria Schönrogge, Tobias Radecke, Brigitte Vollmar

**Affiliations:** Institute for Experimental Surgery, Rostock University Medical Center, Schillingallee 69a, 18057 Rostock, Germany

**Keywords:** Solid-pseudopapillary neoplasms, Acinar cell carcinoma, Founder mutations, Cancer immunology, Diabetes, Hyperglycemia

## Abstract

**Background:**

The involvement of Wnt in carcinogenesis and progression of pancreatic cancer is currently intensely discussed. We evaluated activation of the Wnt signaling pathway by using a Wnt reporter mouse strain expressing β-galactosidase under the control of the Axin2 promotor during pancreatitis induced formation of precancerous lesions. We also evaluated activation of Wnt signaling during interaction of pancreatic cancer with the tumor stroma.

**Results:**

Activation of Wnt signaling was observed during acinar-to-ductal metaplasia after chronic as well as acute pancreatitis. Activation of Wnt signaling was also noticed during growth of pancreatic cancer in an orthotopic syngeneic pancreas cancer model. Activation of Wnt signaling was, however, not observed in carcinoma associated fibroblasts, but was detected in few cell clusters inside the tumor. Genetic ablation of Axin2 significantly reduced body weight without having a major impact on blood glucose concentration. However, ablation of Axin2 had no influence on the observed β-galactosidase positive cell clusters or on tumor weight.

**Conclusion:**

These data demonstrate that the Wnt signaling pathway is activated during acinar-to-ductal metaplasia after injury to the pancreas. However these data do not support a major role of Wnt signaling or of Axin2 in carcinoma associated fibroblasts and tumor growth.

**Electronic supplementary material:**

The online version of this article (doi:10.1186/s13578-016-0116-4) contains supplementary material, which is available to authorized users.

## Dear Editor

The expression of Axin2 is induced by canonical Wnt signaling during cancerogenesis but also during many other Wnt-regulated physiological and pathophysiological processes [1]. The introduction of the β-galactosidase gene into the locus of Axin2 in mice, therefore, generated a reporter strain (Axin2^+/lacZ^), which reliably expresses β-galactosidase in cells where Wnt signaling is activated [1]. Axin2 functions as a scaffold protein that facilitates the phosphorylation and thereby the degradation of β-catenin, which is the key protein necessary for canonical Wnt signaling [2]. Thus, Axin2 inhibits the canonical Wnt signaling pathway in form of a negative feedback loop [1]. In Axin2^lacZ/lacZ^ mice, both Axin2 alleles are replaced by β-galactosidase. This can result in prolonged Wnt signaling in vivo [3].

It is well established that Wnt signaling is involved in the cancerogenesis of multiple gastrointestinal carcinomas [4]. In colorectal carcinoma 90 % of all tumors have a mutation in a gene coding for regulatory components of the canonical Wnt signaling pathway such as CTNNB1 (β-catenin) or adenomatous polyposis coli (APC). These mutations result in activation of the canonical Wnt signaling pathway [4]. In hepatocellular carcinoma 3 to 44 % of tumors contain mutations of CTNNB1 and 5 to 25 % contain mutations in AXIN1 resulting in activation of Wnt signaling [4]. Loss of function mutation of AXIN2 can also be found in hepatocellular and colorectal carcinoma [4].

However, it is still controversial how important this signaling pathway is during carcinogenesis of pancreatic cancer [5]. About 24 % of acinar cell carcinomas (ACC) have molecular alterations in the canonical Wnt signaling pathway. Similarly, solid-pseudopapillary neoplasms (SPNs) in the pancreas usually harbor mutations in CTNNB1 [5]. The prognosis of this rare neoplasm is excellent and most patients are cured by surgical resection [5]. To the contrary, the prognosis of pancreatic ductal adenocarcinoma (PDA) is very dismal. Dependent on the study CTNNB1 mutations have been identified in none or very few PDAs [6, 7]. Nevertheless, recent publications suggest that activation of canonical Wnt signaling via alternative mechanisms might contribute to the carcinogenesis of PDA [6, 7]. For example, mutations of RNF43, which can regulate Wnt signaling, were detected in 6–10 % of PDAs [6, 7].

Only few publications exist that suggest that Wnt signaling might also contribute to tumor stroma interaction [8, 9]. This interaction can be based on the expression of distinct Wnts in cancer associated fibroblasts, which promotes tumor progression [8]. Alternatively, expression of Wnts in carcinoma cells can induce Wnt signaling in the desmoplastic reaction, which indirectly promotes tumor aggressiveness [9].

Thus, the purpose of this study was to evaluate the activation of the Wnt signaling pathway in precancerous lesions such as tubular complexes after acinar-to-ductal metaplasia (ADM) and during the tumor stroma interaction of fully established PDA.

## Wnt signaling in tubular complexes

**Fig. 1:**
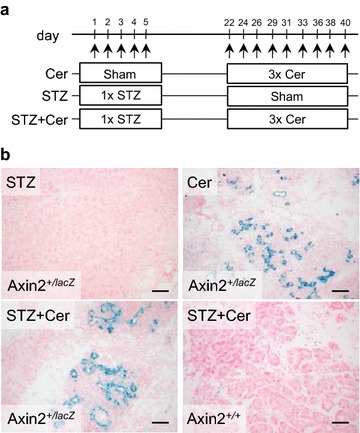
Characterization of the animal model for chronic pancreatitis. **a** Hyperglycemia was induced in two cohorts (STZ, STZ+Cer) by ip injection of 50 mg/kg streptozotocin on day 1–5 and chronic pancreatitis was then induced in two cohorts (Cer, STZ+Cer) by administration of three ip injections of cerulein (50 µg/kg) on the indicated days; the control group (Cer) was treated with the appropriate vehicle solution instead of STZ. **b** β-galactosidase staining in tubular complexes of the pancreas in normoglycemic and hyperglycemic Axin2^+/lacZ^ mice was observed after pancreatitis, whereas no β-galactosidase staining was observed in Axin2^+/+^ mice. *Bars* 50 μm

The formation of tubular complexes was induced by repetitive cerulein administration from day 22 to day 40 of the experimental paradigm (Fig. 1a). Since previous publications demonstrated a detrimental influence of streptozotocin (STZ) induced hyperglycemia on the progression of chronic pancreatitis [10], the pancreas of hyperglycemic mice with chronic pancreatitis (STZ+Cer) was compared to the pancreas of normoglycemic mice with chronic pancreatitis (Cer) and hyperglycemic mice, which had no pancreatitis (STZ). A reliable induction of hyperglycemia in STZ treated mice (STZ: 21.5/18.6–25.0, STZ+Cer: 20.3/16.2–23.0) compared to control mice (Cer: 5.7/4.8–6.2; median/interquartile range in mmol/L on day 22) was noticed. In Axin2^+/lacZ^ mice without pancreatitis no β-galactosidase stained tubular complexes were observed, whereas a strong β-galactosidase staining was observed in tubular complexes during chronic pancreatitis (Fig. 1b; Table 1). No β-galactosidase staining was observed in the pancreas of hyperglycemic Axin2^+/+^ mice during chronic pancreatitis indicating that β-galactosidase staining is dependent on the knock in allele of β-galactosidase into the Axin2 locus (Fig. 1b). Thus, β-galactosidase staining in tubular complexes is not caused by cellular senescence.

**Table 1:** Quantification of β-galactosidase^+^ tubular complexes during chronic pancreatitis on day 40

Axin2^+*/lacZ*^	Number of β-galactosidase^+^ tubular complexes	Number of tubular complexes observed	Number of mice analyzed
Cer	2	2	3
STZ	0	0	3
STZ+Cer	2	4	5

**Fig. 2:**
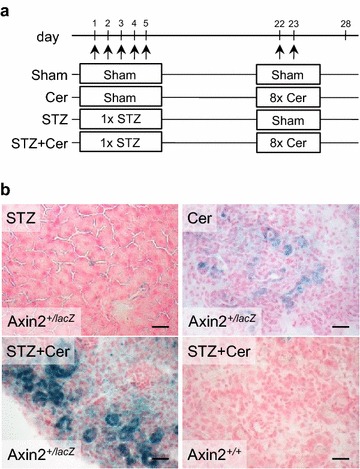
Characterization of the animal model for acute pancreatitis. **a** Hyperglycemia was induced in two cohorts (STZ, STZ+Cer) by ip injection of 50 mg/kg streptozotocin on day 1–5 and chronic pancreatitis was then induced in two cohorts (Cer, STZ+Cer) by administration of eight ip injections of cerulein (50 µg/kg) on day 22 and 23; the control groups (Sham, Cer) were treated with the appropriate vehicle solutions. **b** β-galactosidase staining in tubular complexes of the pancreas in normoglycemic and hyperglycemic Axin2^+/lacZ^ mice was observed after pancreatitis, whereas no β-galactosidase staining was observed in Axin2^+/+^ mice. *Bars* 50 μm

Since only few tubular complexes could be observed during chronic pancreatitis, we also evaluated the formation of precancerous lesions after acute pancreatitis. Acute pancreatitis was induced on day 22–23 in normoglycemic (Cer) or hyperglycemic mice (STZ+Cer) and the pancreas was compared to normoglycemic (Sham) or hyperglycemic mice (STZ) without pancreatitis (Fig. 2a). A strong and reliable induction of hyperglycemia in STZ treated mice (STZ: 21.1/19.5–24.0; STZ+Cer: 22.1/19.4–25.6) compared to control mice (Sham: 5.5/5.2–5.8; Cer: 5.6/5.1–6.1; median/interquartile range in mmol/L) was noticed. Tubular complexes with strong β-galactosidase staining were not observed in the pancreas of sham or STZ treated Axin2^+/lacZ^ mice, but in the pancreas of normoglycemic as well as hyperglycemic Axin2^+/lacZ^ mice during acute pancreatitis (Fig. 2b; Table 2). No β-galactosidase staining was observed in the pancreas of hyperglycemic Axin2^+/+^ mice after acute pancreatitis (Fig. 2b).

**Table 2:** Quantification of β-galactosidase^+^ tubular complexes after acute pancreatitis on day 28

Axin2^+*/lacZ*^	Number of β-galactosidase^+^ tubular complexes	Number of tubular complexes observed	Number of mice analyzed
Sham	0	0	3
Cer	5	6	8
STZ	0	0	5
STZ+Cer	5	5	6

Thus, Wnt signaling is activated in assumed precancerous lesions such as tubular complexes by pancreatitis, which is a well defined risk factor for the development of pancreatic cancer. This observed activation of canonical Wnt signaling upon tissue damage is consistent with previous publications. For example, it was suggested that the observed increase in β-catenin mRNA level [11] or β-catenin protein concentration [12] after tissue damage in the pancreas indicates activation of Wnt signaling. In addition, tissue damage induced the expression of Axin2 as demonstrated by quantitative PCR [12] and the activation of Wnt signaling in ductal cells was also observed after partial pancreatic duct ligation [13].

The following three hypotheses can be defined when describing the observed activation of Wnt signaling during ADM in respect to cancerogenesis: (a) Activation of Wnt signaling has no effect on cancerogenesis, but is a concomitant phenomenon when cells are stuck in dedifferentiation, (b) Wnt signaling inhibits cancerogenesis, (c) Wnt signaling promotes cancerogenesis. Possibly, the function of Wnt signaling is completely different during cancerogenesis leading to PDA or other subtypes of pancreatic cancer.

In respect to cancerogenesis of PDA most data support the first or second hypothesis and disagree with the third hypothesis. For example, activation of Wnt signaling does not cooperate with oncogenic Ras to induce PDA formation in mice [12]. Moreover, only few mutations have been found in human PDAs that activate Wnt signaling suggesting that these mutations are not founder mutations of the carcinoma [6, 7].

In respect to cancerogenesis of SPNs or ACCs most data disagree with the first and second hypothesis and support the third hypothesis. For example, activation of Wnt signaling in mice results in the formation of tumors resembling human SPNs [14], whereas activation of Wnt signaling in p53 deficient mice results in the formation of tumors resembling human ACC [15]. Moreover, human SPNs usually harbor mutations in CTNNB1 that activate Wnt signaling and about 24 % of ACCs in patients show molecular changes in key proteins of the canonical Wnt signaling pathway [5]. Thus, permanent activation of canonical Wnt signaling may promote cancerogenesis of SPNs and ACCs, but not of PDA.

## Wnt signaling during tumor stroma interaction

**Fig. 3:**
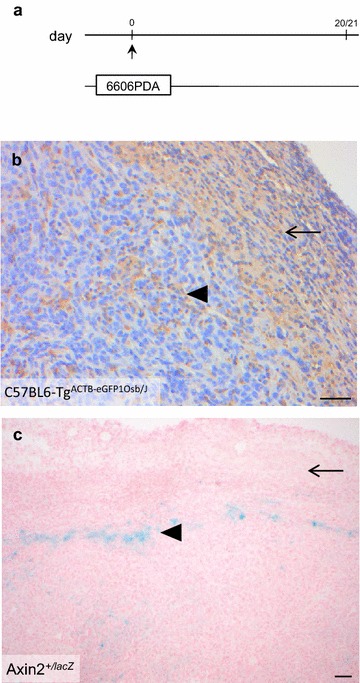
Characterization of the animal model for studying desmoplasia. **a** 6606PDA cells were injected on day 0 into the pancreas of C57BL6-Tg^ACTB-eGFP1Osb/J^, Axin2^+/+^, Axin2^+/lacZ^ or Axin2^lacZ/lacZ^ mice and the tumors were analyzed on day 20 or 21. **b** Desmoplastic reaction visualized by anti-GFP immunohistochemistry in a C57BL6-Tg^ACTB-eGFP1Osb/J^ mouse, which ubiquitously expresses GFP, shows strong GFP expression in fibroblast like cells surrounding the carcinoma (*arrow*), and few GFP expressing cells within the carcinoma (*arrowhead*). **c** Characteristic images of tumors in Axin2^+/lacZ^ mice showing no β-galactosidase staining in fibroblast like cells surrounding the carcinoma (*arrow*), but few clusters of β-galactosidase^+^ cells within the carcinoma (*arrowhead*). *Bars* 50 µm

In order to evaluate, if Wnt signaling can be observed in cancer associated fibroblasts, 6606PDA cells were injected into the pancreas of C57BL6-Tg^ACTB-eGFP1Osb/J^ as well as Axin2^+/lacZ^ mice (Fig. 3a). Carcinomas induced a desmoplastic reaction, which was characterized by GFP^+^ fibroblast-like cells surrounding the carcinoma and a few GFP^+^ cells inside the carcinoma (Fig. 3b). In all Axin2^+/lacZ^ mice fibroblast like cells surrounding the carcinoma did not express β-galactosidase (Fig. 3c). β-galactosidase positive cells were however noticed in about 28 % of tumors in ring like clusters within the tumor (Fig. 3c). In order to evaluate if these cells are dependent on a functional Axin2 gene, we injected 6606PDA cells into the pancreas of Axin2^lacZ/lacZ^ mice. These characteristic clusters of β-galactosidase positive cells were also observed in Axin2^lacZ/lacZ^ mice (Additional file 1). Thus Axin2 is not necessary for the formation of these β-galactosidase positive cell clusters. Since Wnt signaling has been reported to be important at various stages of T cell development [16], these cells might be immune cells migrating into the carcinoma. However, these data do not support the hypothesis that Wnt signaling is activated in carcinoma associated fibroblasts.

**Fig. 4:**
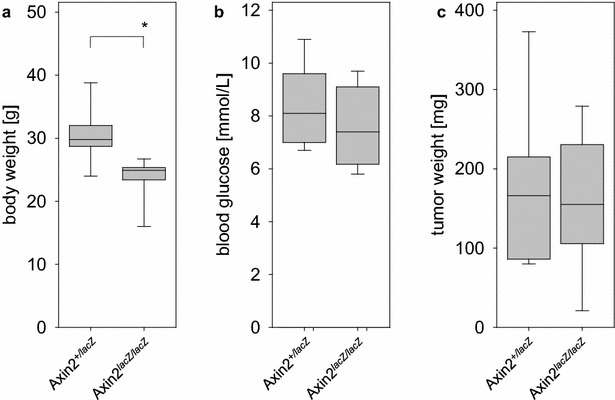
Analysis of body weight, blood glucose and tumor weight in Axin2^+/lacZ^ or Axin2^lacZ/lacZ^ mice. **a** Axin2^lacZ/lacZ^ mice have a significantly decreased body weight. **b** No major influence of Axin2 genotype on blood glucose concentration was observed. **c** No significant influence of Axin2 genotype on tumor weight could be demonstrated. Significant difference: *P = 0.006

In order to evaluate if Axin2 influences the growth of pancreatic cancer we compared the tumor weight in Axin2^lacZ/lacZ^ to the tumor weight in Axin2^+/lacZ^ or Axin2^+/+^ mice. Loss of Axin2 function significantly reduced the body weight without having a major influence on the blood glucose concentration (Fig. 4a, b). We did not observe a major difference in the body weight (Axin2^+/lacZ^: 29.8/28.7–32.1; Axin2^+/+^: 29.7/27.3–34.3 median/interquartile range in g) or in blood glucose concentration (Axin2^+/lacZ^: 8.2/7.4–8.6; Axin2^+/+^: 8.8/7.0–9.9 median/interquartile range in mmol/L) between mice lacking one or none allele of Axin2. A blood cell count did not reveal major differences in the number of leukocytes (Axin2^+/+^: 4.2/3.1–5.9; Axin2^+/lacZ^: 6.0/3.6–6.7; Axin2^lacZ/lacZ^: 6.9/5.3–9.3), lymphocytes (Axin2^+/+^: 2.5/1.9–4.0; Axin2^+/lacZ^: 3.9/2.2–4.7; Axin2L^lacZ/lacZ^: 3.8/2.2–4.7), or monocytes plus neutrophil granulocytes (Axin2^+/+^: 1.7/1.3–2.0; Axin2^+/lacZ^: 2.1/1.4–2.5; Axin2L^lacZ/lacZ^: 3.2/1.5–4.7 median/interquartile range in 10^9^ cells/L) dependent on the mouse genotype. We did also not observe an obvious influence of Axin2 function on the tumor weight when comparing tumors grown in Axin2^+/lacZ^ to tumors grown in Axin^lacZ/lacZ^ (Fig. 4c). We did also not observe a major difference in tumor weight (Axin2^+/lacZ^: 166/86-215; Axin2^+/+^: 283/125–382 median/interquartile range in mg) between mice lacking one or none allele of Axin2. Thus, no major function of Wnt signaling on the growth of pancreatic cancer could be observed in this syngeneic orthotopic animal model.

## Additional file

10.1186/s13578-016-0116-4 In the Supplemental Material Section results from beta-galactosidase stainings, blood glucose concentrations, body weights, tumor weights, as well as number of leucocytes, lymphocytes and monocytes plus ganulocytes are presented.

## Abbreviations

ACC: acinar cell carcinoma
ADM: acinar-to-ductal metaplasia
BrdU: 5-bromo-2’-deoxyuridine
H/E: haematoxylin/eosin
PDA: pancreatic ductal adenocarcinoma
SPN: solid-pseudopapillary neoplasm
